# Lateralization and Time-Course of Cortical Phonological Representations during Syllable Production

**DOI:** 10.1523/ENEURO.0474-22.2023

**Published:** 2023-10-05

**Authors:** Andrew Meier, Scott Kuzdeba, Liam Jackson, Ayoub Daliri, Jason A. Tourville, Frank H. Guenther, Jeremy D. W. Greenlee

**Affiliations:** 1 Department of Speech, Language, and Hearing Sciences, Boston University, Boston, MA 02215; 2 Graduate Program for Neuroscience, Boston University, Boston, MA 02215; 3 College of Health Solutions, Arizona State University, Tempe, AZ 85004; 4 Department of Biomedical Engineering, Boston University, Boston, MA 02215; 5 Department of Radiology, Massachusetts General Hospital, Boston, MA 02215; 6 Picower Institute for Learning and Memory, Massachusetts Institute of Technology, Cambridge, MA 02215; 7 Department of Neurosurgery, University of Iowa Hospitals and Clinics, Iowa City, IA 52242

**Keywords:** audition, clustering, electrocorticography, hemispheres, motor control, speech

## Abstract

Spoken language contains information at a broad range of timescales, from phonetic distinctions on the order of milliseconds to semantic contexts which shift over seconds to minutes. It is not well understood how the brain’s speech production systems combine features at these timescales into a coherent vocal output. We investigated the spatial and temporal representations in cerebral cortex of three phonological units with different durations: consonants, vowels, and syllables. Electrocorticography (ECoG) recordings were obtained from five participants while speaking single syllables. We developed a novel clustering and Kalman filter-based trend analysis procedure to sort electrodes into temporal response profiles. A linear discriminant classifier was used to determine how strongly each electrode’s response encoded phonological features. We found distinct time-courses of encoding phonological units depending on their duration: consonants were represented more during speech preparation, vowels were represented evenly throughout trials, and syllables during production. Locations of strongly speech-encoding electrodes (the top 30% of electrodes) likewise depended on phonological element duration, with consonant-encoding electrodes left-lateralized, vowel-encoding hemispherically balanced, and syllable-encoding right-lateralized. The lateralization of speech-encoding electrodes depended on onset time, with electrodes active before or after speech production favoring left hemisphere and those active during speech favoring the right. Single-electrode speech classification revealed cortical areas with preferential encoding of particular phonemic elements, including consonant encoding in the left precentral and postcentral gyri and syllable encoding in the right middle frontal gyrus. Our findings support neurolinguistic theories of left hemisphere specialization for processing short-timescale linguistic units and right hemisphere processing of longer-duration units.

## Significance Statement

Articulating speech requires control and monitoring of motor outputs that change at timescales ranging from milliseconds to whole sentences. During syllable repetition, we examined how the neural processing of differently-sized speech units is distributed in the brain and across different stages of the task. Using direct electrical recordings from human cerebral cortex, we found that larger linguistic units (syllables) are represented more in the right hemisphere while shorter linguistic units (consonants) are represented more in the left hemisphere. Across time, syllables were represented during vocal output, while consonants were represented during speech preparation and after production. These results indicate a hemispheric specialization for distinct sizes of phonological units and that these units are processed at specific timepoints during speech.

## Introduction

Speaking is one of the most complex motor acts that humans perform, with 20–30 phonemes being produced per second in casual speech (Kent, 2000). In addition to controlling the timing and articulation of these elements, the speech production system must program and control the output of larger units, including syllables, words, and sentences. In this study we used human electrocorticography (ECoG) to investigate two aspects of the neural representation of phonological features of different durations. First, we examined whether the right and left hemispheres preferentially process longer or shorter units. Second, we explored the time-course of representation of these different units. We focused on three phonological units: plosive consonants, which are distinguished by acoustic features on the order of tens of milliseconds (Sweeting and Baken, 1982), vowels with durations in the hundreds of milliseconds, and full consonant-vowel-consonant (CVC) syllables.

From early neuroimaging research, it has been proposed that there is hemispheric specialization for processing distinct timescales of linguistic units: the left hemisphere is sensitive to phonemic and acoustic distinctions within a time window of 20–50 ms, while the right hemisphere processes information with a time window of 150–250 ms (Nicholls, 1996; Johnsrude et al., 1997; Poeppel, 2003). Functional connectivity studies suggest that these differences are supported by the right-hemisphere language-processing system integrating inputs from a wider set of regions than the left hemisphere (Gotts et al., 2013; Simonyan and Fuertinger, 2015; Mišić et al., 2018). Lateralization of processing at these timescales, corresponding approximately to consonant distinctions and syllables, has been demonstrated predominantly in auditory response and perception studies (Wildgruber et al., 2004; Meyer, 2008; Scott and McGettigan, 2013).

Numerous lines of evidence also suggest that right and left hemispheres use different time windows for processing in speech production. Left hemisphere lesions that do not cause complete aphasia induce dysfunction at the level of individual word or sound production (Kendall et al., 2015; Madden et al., 2017; Ripamonti et al., 2018). Conversely, dysfunction in programming prosodic patterns spanning whole phrases is caused by damage to the right hemisphere (Shapiro and Danly, 1985; Peters et al., 2011; Stockbridge et al., 2022).

Neuromagnetic recordings have shown that speech efference copy, measured through auditory response suppression, is prominent in the right hemisphere for whole words and in the left hemisphere only for subcomponents of words (Ylinen et al., 2015). Neural adaptation to speaking a repeated phoneme was found in left but not right higher-order speech areas (Okada et al., 2018). Suprasegmental features of produced speech, including overall speech rate and amount of linguistic content, were shown to be prominently represented in the right temporo-parietal junction (Alexandrou et al., 2017). In addition to hemispheric lateralization, we used the high temporal resolution of ECoG to examine the temporal patterns of how consonant, vowel, and syllable were encoded in cortical responses. Neuroimaging research has revealed key areas and networks that process speech sequences (Bohland and Guenther, 2006; Peeva et al., 2010; Segawa et al., 2015; Rong et al., 2018). Electrical stimulation mapping during neurosurgery has likewise found cortical loci associated with errors at the level of phonemes or syllables (Leonard et al., 2019). However, the temporal resolution of these methodologies limits studies using them to localization of relevant areas. Recording methodologies with greater temporal resolution, such as ECoG, are required to understand the fine-scale time-course of speech sequence representations.

While prior ECoG studies have examined the encoding of speech characteristics during overt production, most have focused on only one level of linguistic organization, such as sentences (Makin et al., 2020; Komeiji et al., 2022), words (Kellis et al., 2010; Martin et al., 2016), phonemes (Pei et al., 2011; Steinschneider et al., 2011; Toyoda et al., 2014), articulatory kinematics (Chartier et al., 2018; Conant et al., 2018; Chrabaszcz et al., 2019), or acoustic features (Bouchard and Chang, 2014; Dichter et al., 2018). Understanding the neural computations required for speech production requires simultaneous investigation of multiple levels of linguistic organization. Some prior ECoG studies have partially addressed this problem, such as a demonstration that perisylvian cortex responses contained representations of spoken words which could not be accounted for by the encoding of their constituent phonemes (Mugler et al., 2014). It has also been shown that decoding of whole spoken words or phrases from cortical activity can benefit from incorporating information about individual phonemes (Herff et al., 2015; Moses et al., 2019), while other approaches have successfully decoded these larger linguistic units while ignoring the constituent phonemes (Kellis et al., 2010; Makin et al., 2020; Komeiji et al., 2022). These studies have not, however, examined how the cortical representations of different scales of linguistic units temporally progress over the course of speech production.

To address these questions, we analyzed cortical responses from participants during a single-syllable CVC repetition task. A novel unsupervised clustering and trend analysis procedure was developed to sort speech-responsive electrodes into groups based on their activity time-courses. We then performed linear discriminant analysis-based classification to determine which electrodes most strongly represented individual phonological units. We found that longer-duration phonological elements were preferentially right-lateralized. Additionally, encoding of different durations of phonological units had distinct time-courses. Shorter-duration units were preferentially encoded before and after speech production, while longer units were preferentially encoded during speech production.

## Materials and Methods

### Participants

Data were obtained from five neurosurgical patients (four males, 1 female) undergoing surgical treatment of medically intractable epilepsy (details in Table 1). Written informed consent was obtained from all participants. All research protocols were approved by the appropriate Institutional Review Board.

**Table 1:** Clinical, demographic, and experimental information for all subjects

Subject	Handed	Age (years)	Hemisphere	Number of trials	Response	Seizure focus	Sex
S357	90+	37	L	108	1004.2 ± 209.3	L mesial temporal	Male
S362	100+	60	L and R	281	894.7 ± 202.4	R ant middle frontal gyrus, L post inf parietal, I frontal pole	Male
S369	100+	30	R	118	830.3 ± 158.7	R mesial temporal (para-hippocampal gyrus, amygdala, hippocampus, fusiform gyrus)	Male
S372	75+	34	L	144	860 ± 121.2	L temporal pole, parahippocampal gyrus	Male
S376	90+	49	R	133	1027.2 ± 261.1	R Parahippocampal gyrus	Female

“Hemisphere” column indicates the location(s) of implanted ECoG grids. “Response” indicates mean and SD of speech reaction time in milliseconds.

### Experimental design

Participants read aloud orthographic stimuli projected onto a video screen. The stimulus set used in this study consisted of consonant-vowel-consonant (CVC) syllables constructed from the combinations of four consonants (/b/, /d/, /g/, and /ʤ/) and three vowels (/æ/, /i/, and /u/) to generate 12 CVCs: /bæg/, /big/, /bug/, /dæʤ/, /diʤ/, /duʤ/, /gæb/, /gib/, /gub/, /ʤæd/, /ʤid/, and /ʤud/. A brief practice session was used to familiarize participants to the orthographic representation of each stimulus. Each collection period (run) consisted of 72 CVCs grouped into 36 pairs. For each pair, the first CVC was presented on the screen for 1 s, followed by a gap of 1.5 s before the second CVC was presented for 1 s. The time between word pairs was randomly drawn between 3, 4, or 5 s. Participants were instructed to say each stimulus as soon as it was presented; there was no “GO signal” between the reading portion and the speaking component. The analyses in the current study used data from only the first word in each pair to minimize potential residual effects from prior productions on the ECoG signal. After an introductory period to familiarize the participant with the experimental protocol, each participant participated in three or four runs containing 36 pairs per run.

### Instrumentation

A condenser microphone (Beta 87C, Shure) captured each participants’ speech, which was amplified (MK3, Mark-of-the-Unicorn) and passed into a multichannel data acquisition system (DAS; System3, Tucker Davis Technologies, or Atlas, Neuralynx) that also simultaneously collected TTL signals denoting presented visual stimuli and ECoG signals. We used an online sampling rate of >12 kHz for voice signals but resampled to 12 kHz offline in MATLAB (MathWorks).

### Electrocorticography acquisition

Research recordings were initiated after the participants had fully recovered from electrode implantation surgery. Participants were awake and sitting comfortably in bed during all experimental recordings. Subdural implantation of the electrode arrays allowed for ECoG signals to be directly recorded from the cortical surface. The ECoG signals were filtered (1.6–1000 Hz anti-aliasing filter), digitized with a sampling frequency of >2000 Hz and then resampled offline in MATLAB.

### Electrode implantation and localization

The devices used to record electrical activity of the brain were a combination of surface (i.e., subdural) and penetrating depth multicontact electrode arrays. Each surface array consisted of platinum-iridium disk electrodes arranged within a silicone sheet (Ad-Tech or PMT). The distance from the center of one electrode to the center of an adjacent electrode measured 5 or 10 mm, while each individual electrode had a contact diameter of 3 mm. Depth electrodes were used in all participants with placement locations dictated by clinical needs of each participant. The extent of the array coverage varied between participants because of the different clinical considerations specific to each participant. After surgical implantation, participants were continuously monitored via video-EEG during a 14-d hospitalization to correlate seizure activity with brain activity for purposes of epilepsy treatment. During this period, high resolution monitoring verified that cortical areas relevant to this study did not show abnormal interictal activity. Once this two-week monitoring period was complete, the electrodes were surgically removed and the localized seizure focus was resected.

High-resolution digital photographs were taken intraoperatively during electrode placement and removal. In addition, preimplantation and postimplantation MR (0.78 × 0.78 × 1.0 mm voxel size) and CT (0.45 × 0.45 × 1.0 mm voxel size) scans were conducted. This information was combined to localize the exact position of the recording electrodes in each participant. FMRIB’s linear image registration tool was used to apply a three dimensional rigid fusion algorithm that successfully allowed preimplantation and postimplantation CT and magnetic resonance imagings (MRIs) to be co-registered (Jenkinson et al., 2002). The coordinates for each electrode from postimplantation MRI volumes were transferred to preimplantation MRI volumes, allowing the relative location of each individual electrode contact in relation to surrounding distinguishable brain structures to be compared in both the preimplantation and postimplantation MRI volumes. This comparison is helpful for improving the accuracy of electrode localization since implantation causes medial displacement of the cerebral hemisphere, which leads to greater deviation of the superficial cortex compared with deeper structures. The resultant electrode positioning was then mapped onto a three-dimensional surface rendering of the lateral surface that was specific to the architecture of each participant’s brain. The estimated spatial error rate when localizing these electrodes is <2 mm.

**Figure 1.**
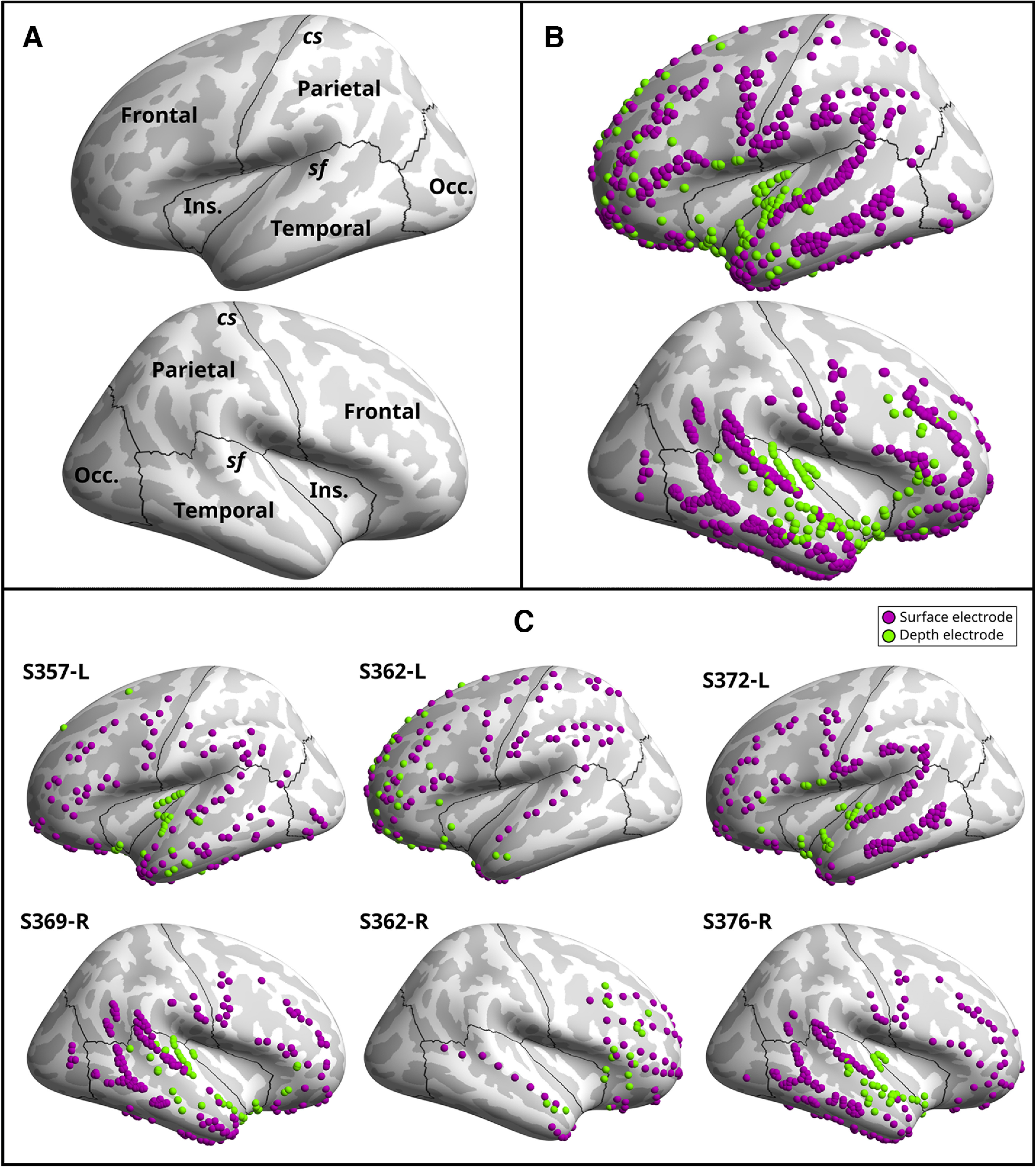
Electrocorticographic recording locations on inflated cortical surfaces. Marker color indicates surface electrocorticography (violet) and depth stereo-electroencephalography (green) electrodes. Depth electrodes were projected to the nearest cortical surface. ***A***, Reference brain template with cortical lobes indicated. Central sulcus (cs) and sylvian fissure (sf) denoted. ***B***, Electrodes from all participants plotted together on common brain. ***C***, Individual participant electrode locations.

Electrode locations are provided in Figure 1, with all electrodes across all participants plotted on the FreeSurfer (Fischl, 2012) common reference brain (Fig. 1*B*) and individual participant electrode locations plotted on the participant’s own magnetic resonance imaging (MRI) scan (Fig. 1*C*). A total of 1036 electrodes were analyzed across the 5 participants.

### Audio preprocessing

Speech onset was measured using a semi-automated method. A 20-ms rectangular kernel was convolved with the absolute value of the recorded audio signal. Resulting values that were above an empirically determined threshold were marked as periods of voicing. Coarse onset estimates were determined to be at the beginning of any contiguous period that exceeded the threshold and with a gap >300 ms from the previous onset. Manual verification and correction was performed using the Praat software suite (Boersma and Van Heuven, 2001) to determine onset validity and refine the location of voicing onset. Average audio signals were computed to get sound envelopes by taking the root mean squared (RMS) value of the raw audio over 50-ms time windows (Kubanek et al., 2013).

### Neural signal preprocessing

The recorded data were downsampled to 1 kHz for further processing with a polyphaser antialiasing filter using the MATLAB ‘resample’ function. After downsampling, the DC component was independently removed for each electrode by subtracting the average value for the channel over the entire collection period. Line noise was removed using notch filters at 60 Hz and its harmonics, using the *tmullen_cleanline* function in EEGLAB (Bigdely-Shamlo et al., 2015), which builds on the Chronux toolbox.

Next, bad channels were identified and removed from further analyses. Bad channels consisted of two types: (1) those that were clinically or experimentally determined to be invalid due, for example, to muscle activity artifacts, and (2) those that were labeled invalid during preprocessing. For the latter, a kurtosis analysis was performed to remove channels that were corrupted by noise or were unexpectedly peaky, such as with eye blink artifacts (Mognon et al., 2011; Tuyisenge et al., 2018). Channels identified for removal were manually verified. Signals were then re-referenced according to a common average reference scheme (Crone et al., 2001), with electrodes averaged across each grid of electrodes to remove non-neural noise artifacts from the shared collection hardware.

We focused on our analysis on high γ power, which represents spiking and dendritic potentials of local neurons (Ray and Maunsell, 2011; Dubey and Ray, 2019; Leszczyński et al., 2020), particularly those in cortical layer 3 (Dougherty et al., 2019). Specifically, the log-analytic amplitude of the Hilbert transform was used to bandpass filter the ECoG recordings into 8 logarithmically spaced bands spanning 70–150 Hz (Moses et al., 2016). The analytic signal was computed for each band and the absolute value was taken as the analytic amplitude, which represents the envelope of the bandpass filtered signal. These amplitudes were then averaged together to get a log-analytic amplitude representation.

After filtering, responses for each trial were aligned to voice onset. Extracted trial epochs had a 3-s duration, starting 2 s before voice onset and ending 1 s after, with average stimulus presentation 916 ms before onset. A baseline period before stimulus presentation was used to normalize the signal. The baseline period was taken to be the first 500 ms before stimulus presentation, and the high γ signal at each time point in the trial was re-referenced as a z-score relative to the trial’s baseline period (Edwards et al., 2010). All trials were averaged to create an event-related spectral perturbation (ERSP; Makeig, 1993) that captures the average response of high γ power.

Next, electrodes that had a significant ERSP response were identified and kept for further analysis. First, electrodes that did not deviate beyond a 95% confidence interval of baseline activity were marked as nonsignificant and removed from further analysis. Remaining electrodes were then subjected to a Kalman filter-based trend analysis (see below, Trend analysis). Only electrodes that deviated from the data-driven baseline trend identified by the Kalman filter were kept for further analysis. The 1036 total electrodes from the five participants were reduced to 334 significant electrodes, of which 163 were in the left hemisphere and 171 were in the right hemisphere.

### Clustering of speech onset-locked responses

Clustering was performed to generate electrode groupings using pairwise distances. Pairwise comparisons of ERSP responses (after normalizing each ESRP response to range from 0 to 1) were computed using a distance measure that emphasized activity differences further away from the nontask baseline. Specifically, an exponential difference between signal values was computed using the following equation:

$$DIST=\sqrt{\sum_{i}{\left(e^{pi}-\;e^{qi}\right)}^{2}},$$

where π and qi are the signals on electrodes p and q at time point i. This distance measure emphasizes significant activity time points and hence puts more weight on the similarities or differences of these time points. This differs from most prior studies, which characterized electrode signal similarity using linear measures (correlations) that put equal weight on nonsignificant time points rather than focusing on similarities or differences during key time points of the activity such as peaks or plateaus.

A hybrid clustering method that combines partitioning and hierarchical clustering was used to identify electrodes that displayed similar time-courses according to the distance measure just described (Liao, 2005; Aghabozorgi et al., 2015). This approach initially assigns each electrode to its own cluster, as in hierarchical agglomerative clustering (see Extended Data Fig. 2-1). At each iteration step the pairwise distance between each cluster is computed. The two clusters with the closest match are then merged. Merging consists of re-computing an average for all electrodes that are members of the cluster, which results in a new cluster centroid. This hierarchical approach by itself creates a nonmonotonic cluster tree. To ensure a monotonic cluster tree, a partitioning refinement step is taken to look at any cluster that has a closer distance measure in the new cluster representations compared with the distance measure of the clusters just merged. The partitioning step reallocates electrodes between the two merged clusters and any clusters breaking the monotonic relationship, generating clusters that maintain a monotonic cluster tree. This process repeats for each step of the iterative clustering until all electrodes are merged into a single cluster. This clustering method combines the strength of cluster tree generation through hierarchical clustering methods with the ability to maintain a monotonic cluster tree to enable the selection of the number of clusters. The partitioning refinement step functions similar to k-means over a subset of the electrodes.

After this clustering procedure, the number of clusters that best capture the true nature of the underlying data were selected. A distance threshold can be set to select the number of clusters from the cluster tree. Since there is no clear method for choosing a threshold, two different methods were employed to choose the most informative number of clusters. First, the “elbow” method (Thorndike, 1953) was used to select the number of clusters based on the elbow in the cluster tree, which evaluates the distances between clusters at each branch of the tree. These distances across different numbers of clusters are plotted in Extended Data Figure 2-1. We selected the elbow from the derivative of the distance function, which was more pronounced (indicated by the red circle in Extended Data Fig. 2-1). This elbow, where the derivative shows a very noticeable slowing rate of change in the reduction in distance that additional clusters would add, occurred at six clusters.

In the second method for determining an optimal number of clusters, the percent variance explained using a comparison of the sum of squares of within-cluster variance to total variance was calculated (Goutte et al., 1999). This method also indicated that six clusters provided the best account of the data. This choice of clusters explains 69% of the variance, with additional clusters only marginally adding to the explained variance. Because both metrics suggested the same optimal number of clusters, six clusters were used for all subsequent analyses.

### Trend analysis

A novel data-driven statistical method was used to identify trends and change points in high γ traces for two purposes: (1) to identify electrodes in which activity changed significantly from the baseline, and (2) to quantitatively describe the shapes of the characteristic time-courses resulting from the cluster analysis (Kuzdeba et al., 2019). Past studies have used functional representations to capture changes in neural temporal dynamics, such as splines (Brumberg et al., 2015), which provide piecewise linear breakdowns for trend analysis. We instead used a dynamic method that detects trend changes in the data with fewer priors on the form the changes can take. The method is based on detecting change points (Page, 1963; Aminikhanghahi and Cook, 2017) with a Kalman filter (Kalman and Bucy, 1961). A Kalman filter is a statistical method that estimates the internal state of a linear dynamic system from a series of measurements that include process noise (in our case, noise inherent to the neural signals) and observation noise (noise inherent to the ECoG recording process).

For our trend analyses, the Kalman filter estimates high γ power (g) and its time derivative (or trend, $\dot{g}$), represented by the two-dimensional state vector X = [g, $\dot{g}$^T^, where ^T^ is the transpose function. The state transition matrix, ***A***, captures the relationship between these states at each time point *i* and is used to generate an initial prediction of ***X***(:,*i*) based on the prior time point:

$$\begin{array}{l} \;\;\;\;\;\;\;A=\left[\begin{array}{c} 1\\ 0 \end{array}\begin{array}{c} 1\\ 1 \end{array}\right]\\ X\left(:,i\right)=AX\left(:,i-1\right) \end{array}.$$

Here we used a simple linear estimation procedure. More complex filters were tested, but the linear state transition matrix performed best and was the most parsimonious. The covariance (or uncertainty) of the estimate, termed ***P***, is calculated using the equation:

$$P\;=\;APA^{T}+\;Q,$$

where ***Q*** is the covariance of the process noise, i.e., the noise present in the underlying neural activity. For the first time step of the baseline period, ***X*** is initialized to *[0 0]^T^*, ***Q*** is initialized to difference-stationary whitened variance during the baseline period, and ***P*** is initialized to ***Q***. Together the calculations above are called the prediction step.

After predicting the state of the system based on its prior estimated state in the prediction step, the Kalman filter then updates this estimate based on the observation *Z(1)*, which is the high γ power measured by the electrode at the current time point. The rate of change of the measured power is also estimated as the difference between the power at the current time point and the power at the last initialization point divided by *Δ*, which is the number of time steps since the last initialization point. The update step is governed by the following equation:

X(:,i)=X(:,i) + K(Z(i)−HX(:,i)),

where K is the Kalman gain that determines the relative weight to put in new observations versus the prediction, and H is the measurement model that maps the model state space X into the observation space Z. In our case H is set to [1 0]^T^ since only the power is observed. The Kalman gain is calculated as follows:

$$K=\alpha PH^{T}{\left(HPH^{T}\;+\;R\right)}^{-1},$$

where R is the covariance of the observation noise, which is initialized to be the overall variance in the power of the baseline period, and α is a decay factor that gives less weight to new observations as time persists and evidence is gained for a given trend according to the following equation:

$$\alpha =e^{-\Delta /\left(\delta F_{s}\right)},$$

where Fs is the sampling rate and δ is a time constant set to 100 ms. The parameter *α* functions to “freeze” trends as evidence for the trend accumulates, which in turn allows deviations from the trend (change points) to be identified before the model is corrupted by data that does not fit the trend.

To identify change points, a threshold is set for how far away a new observation, Z*(1)*, can be from its estimate, X(1,*i*). The threshold is set based on the empirical variance across the electrode’s z-scored baseline period and only takes into account the power term. An inverse Q-function is used to get the 95% confidence value for the variance in the baseline power for each electrode. This results in a β distribution across all electrode confidence values, which are all z-scored to have the same statistical representation. A more stringent 99% confidence value is used to select the threshold to use from this distribution, resulting in a threshold that is representative across all electrodes and not electrode-specific, and thus correcting for multiple comparisons. If a significant change in trend is detected at any point after the baseline period, the electrode is deemed to be responsive to the task and is included in the cluster analysis.

Each time a change point is detected, a new Kalman filter is initialized similar to the original one started on the baseline period, but now the data used to initialize the filter is from the time of the change. Values are re-initialized from priors or what was found in the baseline period, as discussed above, with two exceptions. First, the estimate covariance, ***P***, is recalculated using the current values at the time point of the signal, as prior studies have found that there are changing dynamics during an ECoG task that are dependent on the activity being captured, such as a reduction in variability during stimulus onset (Dichter et al., 2016) and an increased variance with increasing response amplitudes (Tolhurst et al., 1983; Ma et al., 2006). Second, the empirical trend is recalculated using 100 ms of data around the change, with 10% of points in the past and 90% in the future of the change. The decay rate is reset to allow the Kalman filter time to re-learn the new trend before it becomes “frozen.”

### Classification analysis

In order to study the functional roles of recording sites, we employed a linear discriminant analysis (LDA)-based machine learning approach for decoding stimulus characteristics from physiological responses, using a similar procedure as previously published (Lotte et al., 2015). Three separate analyses were performed for each electrode to test its representation of the spoken syllables’ consonant pair, vowel, or whole-syllable identity. Consonant order was taken into account, so that four consonant labels were classified (/b-g/, /g-b/, /d-ʤ/, and/ʤ-d/). For each participant, classification was performed by first dividing all trials into five nonoverlapping subsets. Four sets were used as training data and the fifth was used as test data. A feature set was created by dividing each electrode’s HG power response in the 2 s around voice onset into 50-ms moving averages with 25-ms step size, creating 79 possible features per electrode. Feature selection was performed on the training data using minimum redundancy maximum relevance (mRMR; Peng et al., 2005). The top 150 features across all electrodes were selected for use in classification. Five-fold cross-validation was conducted by training the LDA classifier with the data from the training set, then determining percentage classification accuracy on the test set. This procedure was repeated five times, using each data fold as the test set.

To compute a classification strength metric for each electrode, the classification procedure was repeated while leaving electrode out of the analysis. That electrode’s classification strength score was computed as the accuracy when using all electrodes minus the accuracy when that electrode was excluded. The electrodes most strongly encoding a stimulus characteristic (consonant, vowel, or syllable) were then selected as the 30% of electrodes across all participants with the highest classification strength score. For each cluster, the percentage of electrodes of that cluster in the top 30% of classification for a stimulus label was computed. For the right and left hemispheres, the proportion of top-30% electrodes in that hemisphere was computed and compared against the chance level, taking into account the total number of responsive electrodes in each hemisphere.

To assess whether the classification procedure was successful in decoding phonemic labels at an above-chance rate, we compared decoding rates to chance levels computed to account for number of trials used. The following formula using a cumulative binomial distribution (from Combrisson and Jerbi, 2015) was used:

$$P\left(z\right)=\overset{n}{\underset{i=z}{\sum }}\;\left(n\;i\;\right)\times {\left(\frac{1}{c}\right)}^{i}\times {\left(\frac{c-1}{c}\right)}^{n-1},$$

where *c* is the number of unique labels (four for consonant, three for vowel, 12 for syllable), *n* is the total number of trials analyzed, and *P(z)* is the probability of achieving at least z correct classifications by chance. For each subject and each of the 3 phonemic units, a vector was generated containing binary values, with each binary value indicating whether that phonemic unit was classified correctly on a given trial (using responses from all electrodes in that subject). These trial vectors from all 5 subjects were then concatenated into a 646 × 1 vector, to represent decoding accuracy for a phonemic unit across all trials in the dataset. Above-chance decoding for each phonemic unit was considered to be attained if accuracy across all trials exceeded the chance level at *p* = 0.05. These chance levels were 27.8% for consonant, 36.4% for vowel, and 10.2% for syllable. A *p*-value was computed for the accuracies obtained for each phonemic unit using the same method.

In addition, we used this methodology to compute above-chance decoding accuracy for individual subjects, taking into consideration the number of trials each subject performed. A subject was considered to have significant decoding accuracy for a given phonemic unit if classification was above chance at *p* = 0.05.

### Comparison of response analysis windows used in classification

We compared our initial classification procedure, using electrode responses from the 2-s window surrounding speech onset, to a procedure using windows corresponding to the response width of individual clusters. The purpose of this alternative analysis was to determine whether classification accuracy would be higher if the decoding procedure used only data from timepoints when each cluster was most active. For each cluster, the speech onset-locked time window between “start” and “end” times listed in Table 2 was used. In all cases, this cluster-specific window was shorter than the 2-s epoch used in the initial classification analysis. As with the fixed-window classification, the response window was divided into 79 features of equal duration, with a stride length equal to half of the feature duration. The same classification procedure, including mRMR feature selection and fivefold cross-validation, was conducted using all electrodes from a given subject with these cluster-specific windows. For consonant, vowel, and syllable, we then compared subject-level decoding accuracy in the fixed-window procedure to that in the cluster specific-window procedure with paired *t* tests.

**Table 2:** Cluster timing landmarks and activity slopes

Cluster	Start (ms)	Onset (1/s)	Peak (ms)	Offset (1/s)	End (ms)
C1	−1040	1.16	−480	−1.08	320
C2	−830	1.27	−340	−0.70	800
C3	−710	0.72	200	−0.69	>1000
C4	−380	1.13	110	−1.2	640
C5	−50	2.07	200	−1.1	680
C6	10	1.35	570	−1.33	>1000

“Start” and “end” columns indicate times of deviation from and return to baseline high γ power, as derived by trend analysis (see Materials and Methods). “Onset” and “offset” columns indicate the slope of high γ power during the corresponding periods. “Peak” column indicates the time of maximum response magnitude. All times are relative to speech onset.

### Preferential encoding of phonemic units in single electrode responses

In addition to our analysis of the top-encoding electrodes for each phonemic unit, we performed a separate decoding analysis to find cortical sites that preferentially encode either consonant, vowel, or syllable. This analysis was different from the previously described classification procedure in two respects. First, this analysis focused on electrodes which show a strong preferential encoding for one of the three phonemic units over the other two (for example, stronger encoding of vowel identity than consonant and syllable identity), rather than focusing on the strongest-encoding electrodes for a single phonemic unit, regardless of the other two phonemic units. Second, a separate classification procedure was run on responses from each individual electrode, rather than responses from all electrodes’ simultaneous responses, or all electrodes minus one. Thus, this analysis investigated preferential encoding of a phonemic unit at a specific cortical location, rather than the contribution of activity at that recording site to speech encoding within a broader cortical network.

For each electrode, the same LDA-based machine learning procedure was performed as described above, but using only response features from that electrode. mRMR was used to select the top 10 out of the total 79 features from each electrode for classification. As with subject-level classification, features were taken from a 2-s window centered on speech onset. A classification result was generated for each trial, which was compared with the actual phonemic label to mark it as correct or incorrect for a given electrode. Cluster identity was not considered in this analysis.

A bootstrap hypothesis testing procedure was performed in which a distribution of mean accuracies was generated from a randomly resampled set of trials. For each subject, 10,000 random sets of trial indices were generated, where each set contained the number of usable trials from that subject, with replacement. These 10,000 trial sets were used for all electrodes within a subject. For each of these sets, the mean classification accuracy of this electrode across all trials in this set was computed for consonant, vowel, and syllable. This procedure generated sets of 10,000 mean accuracy values, with a separate distribution for each of the three phonemic units. Because chance-level accuracy differed across phonemic units, a normalization step was required to compare across units. After accuracy distributions were generated for all electrodes of a subject, these electrodes were compared for each trial set. Within a given trial set, each electrode was given an accuracy rank for each phonemic unit, where the least accurate electrode received a rank of 1 and the most accurate electrode received a rank of N, where N equaled the number of usable electrodes within this subject.

Next, a phonemic preference index (PPI) value was computed for each of the 3 phonemic units, which served as a metric of preferential encoding of this unit in this trial set. The PPI for a phonemic unit was computed as the greater of the following two values:

$${\text{Rank}}_{\text{this}\_\text{unit}}-{\text{ Rank}}_{\text{other}\_\text{unit}\_\text{1}}$$

$${\text{Rank}}_{\text{this}\_\text{unit}}-{\text{ Rank}}_{\text{other}\_\text{unit}\_\text{2}},$$

where Rank_this_unit_ was the mean accuracy of this electrode on this set of trial for this phonemic unit, and Rank_other_unit_1_ and Rank_other_unit_2_ were the corresponding accuracy ranks of the other two phonemic units. For each phonemic unit, a distribution of 10,000 PPI values per electrode were analyzed. Electrodes for which the 5th percentile of lowest PPI values from this distribution for a phonemic unit was above zero were considered to preferentially encode that phonemic unit. Electrodes with significant PPI were plotted on an inflated cortical surface, with color labels indicating whether consonant, vowel, or syllable was preferentially encoded. If an electrode showed significant preferential encoding for two phonemic units, the phonemic unit with higher mean PPI was used to determine its plotted marker color.

### Code accessibility

Code used for data analysis can be found in the repository at https://github.com/GuentherLab/ecog-clust-paper-code.

## Results

### Canonical clusters

**Figure 2.**
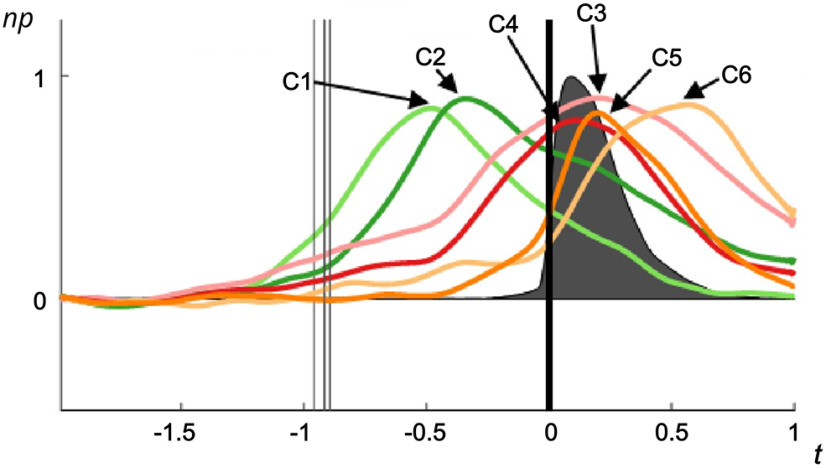
Time-courses of six characteristic responses of electrode clusters during syllable production aligned to speech onset. Responses were quantified as normalized high γ power (np), shown on *y*-axis. Average audio signal amplitude indicated by gray. Clusters were determined from individual electrode time-courses using a novel partitioning/hierarchical clustering procedure (see Extended Data Fig. 2-1).

**Figure 3.**
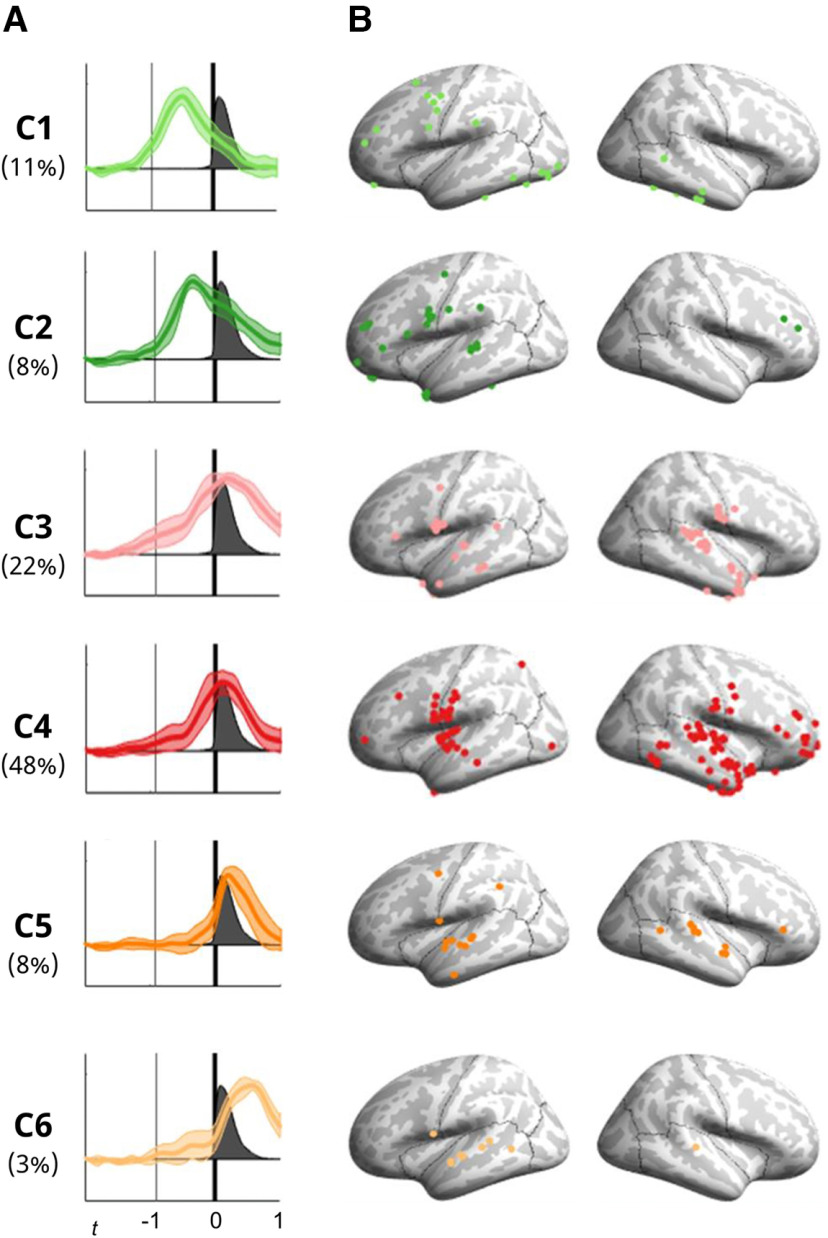
Voice-aligned cluster time-courses and cortical locations from all participants combined. ***A***, Characteristic cluster time-courses shown as dark colored lines with SEM at each time point in light colored shading. Vertical thick black lines show voicing onset. Percentages indicate the proportion of all responsive electrodes falling into each cluster. ***B***, Locations on cortex of electrodes within each of the six clusters. Depth electrodes are projected onto the nearest cortical surface.

High γ responses during cued CVC syllable production fell into six clustered time-courses, which were numerically labeled according to their response onset time (Fig. 2). Clusters are presented with mean characteristic time-courses and SEM (Fig. 3*A*) alongside the location of all electrodes within the cluster from all participants (Fig. 3*B*). Clusters C1 and C2, comprising 19% of electrodes, had activity peaks 1000–300 ms before voice onset, before significantly declining in activity before voicing (Table 2). These electrodes were mostly in left frontal cortex and posterior inferior temporal cortex. Clusters C3 and C4 had activity peaks centered on speech production and were located in bilateral frontal and parietal cortex surrounding the ventral central sulcus, left inferior frontal cortex, right anterior frontal cortex, insula, superior and middle temporal cortex, and to a lesser degree, inferior temporal cortex. C3 (22% of electrodes) had a broad activity time-course, while the activity of C4, nearly half of all electrodes (48%), had a brief response pattern which coincided more specifically with speech production. Cluster C5 (8% of electrodes) had the narrowest activation time-course, with a sharp onset peaking shortly before voice offset. These electrodes fell primarily in the superior temporal gyrus and neighboring cortical regions. Cluster C6, comprising only 3% of electrodes, was active mostly after speech production, peaking 570 ms after voice onset. These electrodes were almost all located on the left superior temporal gyrus.

10.1523/ENEURO.0474-22.2023.f2-1 Extended Data Figure 2-1 Clustering dendrogram schematic and effect of cluster number on mean distance. ***A***, Dendrogram illustration of cluster tree showing at what distance each channel gets merged into a cluster. Moving left to right, each channel is initialized as its own cluster and is iteratively merged until a single cluster remains. Temporal profiles of three example channels are plotted for illustrative purposes to depict when these channels would be clustered together. ***B***, Relationship between number of clusters and the distance needed to result in a tree with that number of clusters. The red marker denotes the location that is selected for providing the number of clusters using the percentage of variance explained, which sets the distance threshold for selection. This point visually aligns with the “knee in the curve” heuristic. Download Figure 2-1, TIF file.

### Lateralization of speech feature encoding

In order to determine how speech features were represented in the activity of responsive electrodes, we performed LDA-based classification of three features of the produced syllables: consonant pair (B-G, G-B, D-J, J-D), vowel (-A-, -I-, -U-), and whole-syllable identity (12 unique syllables). Three separate analyses were performed for these features. For each feature, a classifier was used to decode the speech feature with all electrodes from a participant, using a 2-s response window centered on voice onset. We confirmed that across all trials from all subjects, decoding was significantly higher than chance (consonant: *p* < 10^−10^; vowel: *p* < 10^−9^; syllable: *p* < 10^−7^; *n* = 646 trials; binomial cumulative distribution test; see Materials and Methods). When analyzing classification performance in individual subjects, we found 4/5 subjects showed above-chance (*p* < 0.05) decoding for consonant, 5/5 subjects were above chance for vowel, and 4/5 subjects were above chance for syllable. All subjects showed above-chance decoding for at least two of the three phonemic units.

**Figure 4.**
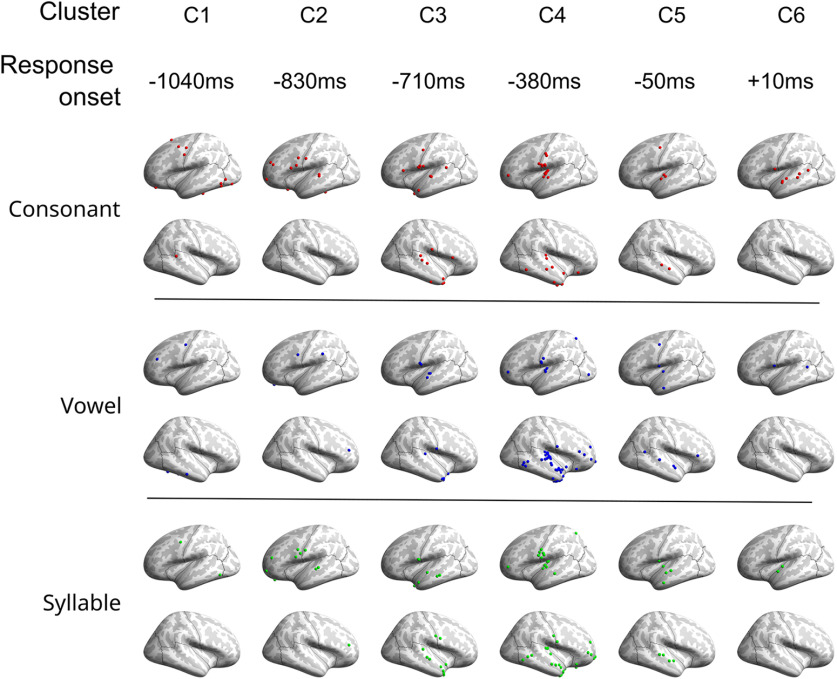
Locations of strongest 30% encoding electrodes for spoken consonant, vowel, and whole-syllable identity. For each phonemic unit, top-encoding electrodes are presented separately depending on their cluster identity. Clusters are organized in columns according to their high γ power onset time aligned to speech onset.

Next, for each electrode, the classifier was rerun with that electrode excluded. That electrode’s classification performance was computed as the classification accuracy with that electrode included minus accuracy with that electrode excluded. Finally, the top 30% of all electrodes in terms of classification performance across all participants were found for each speech feature (*n* = 89 electrodes for each feature). These top-encoding electrodes were then plotted using the standardized MNI-152 template, sorted by cluster and speech feature (Fig. 4).

**Figure 5.**
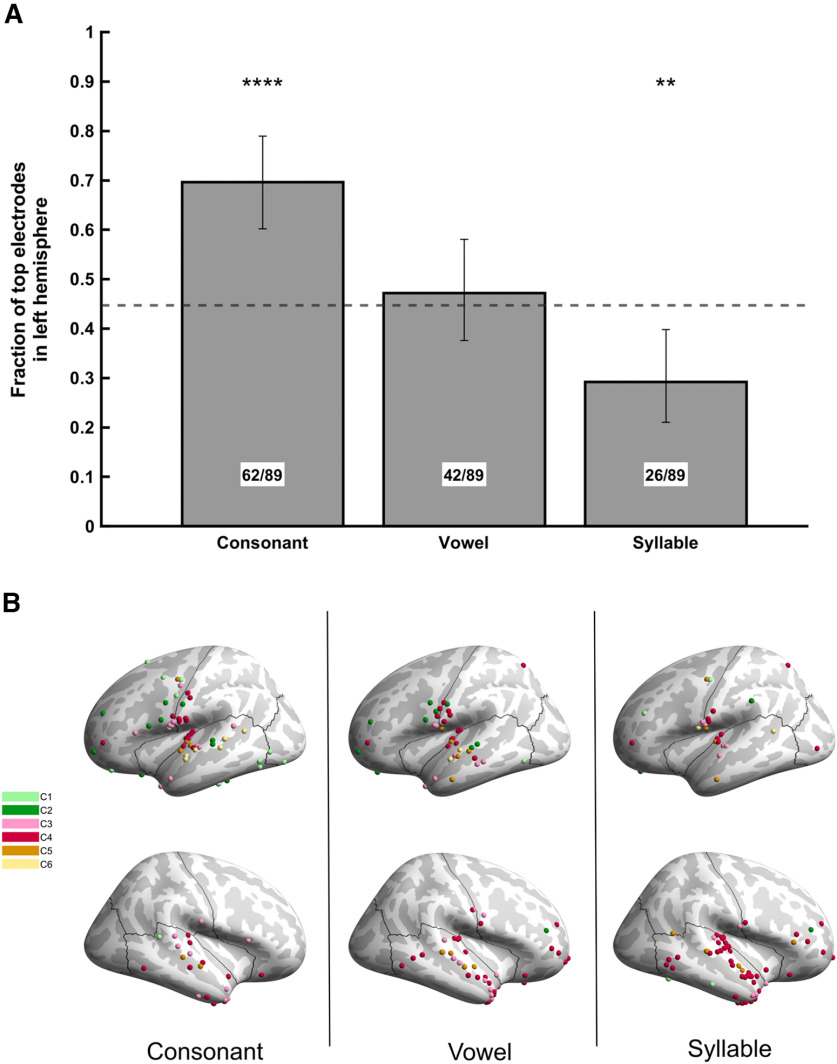
Hemispheric lateralization of top-30% encoding electrodes for consonant, vowel, and syllable. ***A***, Proportions of right-hemisphere electrodes among top-encoding electrodes for each phonemic unit. Error bars show 95% confidence intervals. Dotted horizontal line shows the chance proportion of right-hemisphere electrodes, determined as the proportion of all responsive electrodes in the right hemisphere. Stars indicate deviations from chance proportion (FDR-corrected two-tailed binomial test). *p*-value stars indicate: *p = 0.05, **p = 0.01, ***p = 10^–3^, ****p = 10^–4^. ***B***, Cortical locations of top-30% encoding electrodes from all five participants for consonant, vowel, and syllable. Locations are from Figure 4, with clusters combined onto the same cortical surface. Colors indicate cluster identity of electrodes.

We examined whether there was a hemispheric bias for top-encoding electrodes for each speech feature, relative to the overall proportion of electrodes in each hemisphere (44.7% left hemisphere; Fig. 5). Across all three features, top-encoding electrodes were nonrandomly distributed across hemispheres (*p* < 0.001, χ^2^). Consonant-encoding electrodes were biased to be located in the left hemisphere (69.7% left; FDR-corrected *p* < 10^−4^; two-tailed binomial test). Vowel-encoding electrodes were evenly distributed across right and left hemispheres (47.2% left; FDR-corrected *p* = 0.71; two-tailed binomial test). Syllable-encoding electrodes were biased to be located in the right hemisphere (29.2% left; FDR-corrected *p* < 0.006; two-tailed binomial test).

### Lateralization of encoding in a single subject

In one participant (S362), electrode recordings were acquired in both hemispheres, enabling a within-participant comparison of phonemic encoding. We increased the number of electrodes considered to be top-encoding to the best 60% (*n* = 13 top-encoding electrodes per phonemic unit). This proportion was increased to provide statistical power while still excluding electrodes with negligible or negative contributions to classification. Encoding strengths were normalized within each phonemic unit by dividing encoding strength by the maximum strength of any electrode for that phonemic unit. To investigate the possibility of an influence of hemisphere on phonemic encoding, a two-way ANOVA was run with normalized encoding strength as dependent variable and hemisphere and phonemic unit as independent variables. Results showed no main effect of hemisphere (*p* = 0.15) and a near-significant interaction effect between hemisphere and phonemic unit (*p* = 0.06). The direction of this interaction was generally similar to that seen across all subjects combined, with localization to the left hemisphere increasing consonant encoding strength (interaction coefficient = 0.07) and reducing syllable encoding strength (coefficient = −0.14). However, this subject’s results differed from the all-subjects dataset in showing a similar positive interaction between left-hemisphere localization and vowel encoding (coefficient = 0.07) as consonant encoding, whereas across all subjects, vowel was encoded equally as strongly in both hemispheres (Fig. 5).

### Hemispheric bias of clusters

**Figure 6.**
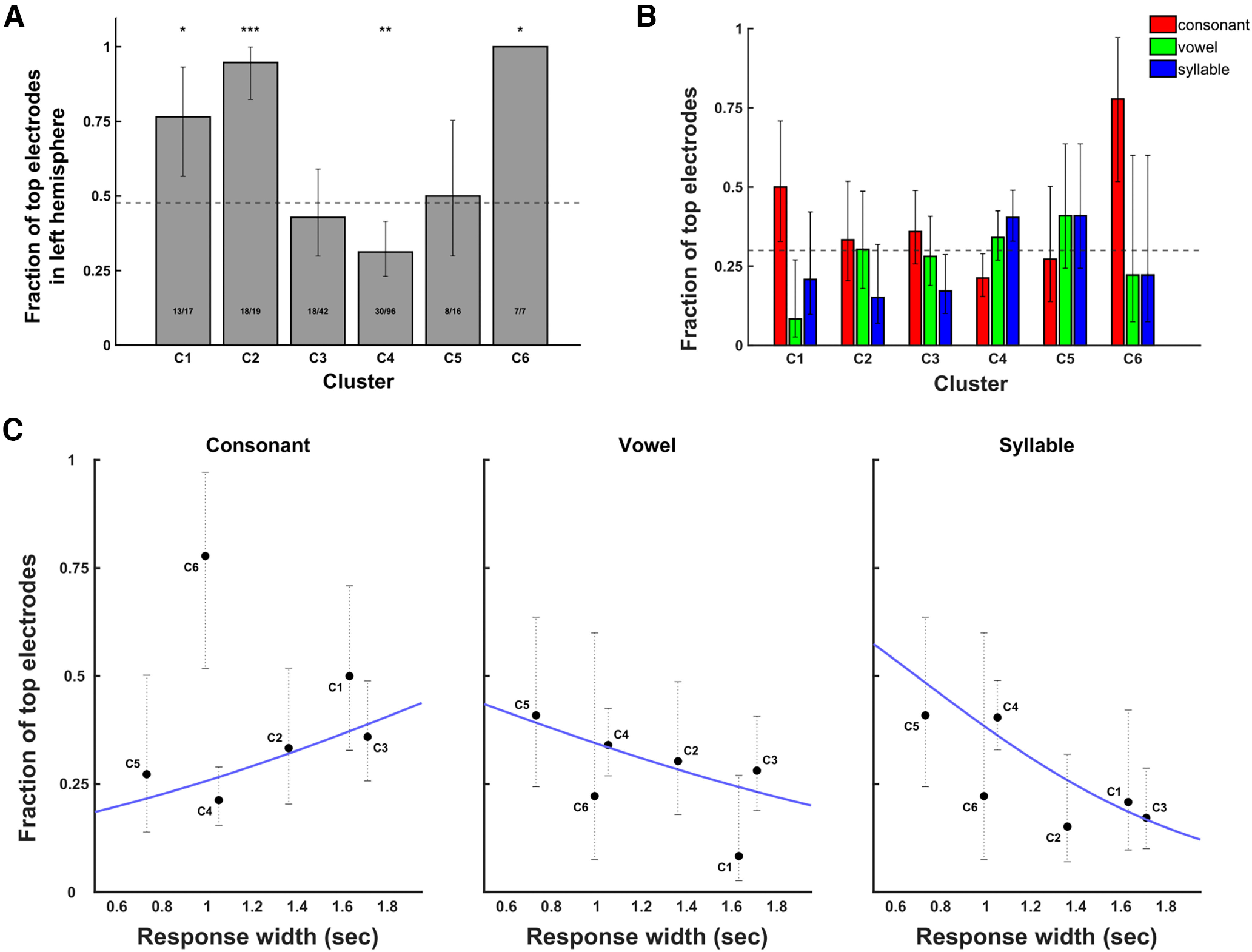
Relationships between cluster lateralization, time-courses, and speech encoding. ***A***, Hemispheric lateralization of clusters, organized in order of high γ power response onset time. Bars show proportions of electrodes in each cluster in the right hemisphere, with error bars showing 95% confidence intervals. Only electrodes that fall into the top 30% of encoding for consonant, vowel, or syllable are included in this plot. (For similar analysis including all electrodes, see Extended Data Fig. 6-1.) Dotted horizontal line shows the chance proportion of right-hemisphere electrodes, determined as the proportion of all top-encoding electrodes in the right hemisphere. Stars indicate deviations of individual clusters from chance proportion (FDR-corrected two-tailed binomial test). *p*-value stars indicate: *p = 0.05, **p = 0.01, ***p = 10^−3^, ****p = 10^−4^. ***B***, Proportion of top-30% electrodes within each cluster encoding consonant, vowel, and syllable. Error bars show 95% confidence intervals. Dashed line indicates 30% chance level. ***C***, Relationship between cluster response width and proportions of electrodes which are top-30% encoders for consonant, syllable, or word. Cluster width is the time between response onset and offset of the cluster’s characteristic response (see Table 2 and Fig. 3). Cluster proportion means and error bars are the same as in panel ***B***. Trendlines were drawn using the logistic regression fit.

We next examined the lateralization of electrodes with respect to cluster. We found that clusters were nonrandomly distributed in each hemisphere (*p* < 10^−6^, χ^2^, *n* = 293 electrodes; Fig. 6*A*). The two earliest-responding clusters were found to be more localized in the left hemisphere (cluster 1: 75% left, FDR-corrected *p* < 0.0052; cluster 2: 94% left, FDR-corrected *p* < 10^−8^; two-tailed binomial test). The cluster with activity most aligned with speech production, cluster 4, was significantly right-lateralized (27.7% left; FDR-corrected *p* < 0.001; two-tailed binomial test). C6, the latest responding cluster, was left-lateralized with only 1 out of 9 electrodes in the right hemisphere (88.9% left; FDR-corrected *p* < 0.026; two-tailed binomial test). This analysis was initially performed while including only those electrodes which were top-encoders for at least one of the three speech features, to investigate lateralization of only those responses most relevant for speech production. We also performed this analysis when including all responsive electrodes, regardless of encoding performance (Extended Data Fig. 6-1). Similar results were found as when including only top-encoding electrodes: cluster lateralization was nonrandom (2; *p* < 10^−3^, χ^2^, *n* = 197 electrodes), and individual clusters showed the same biases for localization to the right or the left hemisphere.

10.1523/ENEURO.0474-22.2023.f6-1 Extended Data Figure 6-1 Hemispheric lateralization of clusters, organized in order of high-gamma power response onset time. Bars show proportions of electrodes in each cluster in the right hemisphere. All responsive electrodes are included in this plot. (For similar analysis of including only top-encoding electrodes, see Fig. 6*A*.) Dotted horizontal line shows the chance proportion of right-hemisphere electrodes, determined as the proportion of all electrodes in the right hemisphere. Stars indicate deviations of individual clusters from chance proportion (FDR-corrected two-tailed binomial test). *p*-value stars indicate: *p = 0.05, **p = 0.01, ***p = 10^−3^, ****p = 10^−4^. Download Figure 6-1, TIF file.

**Figure 7.**
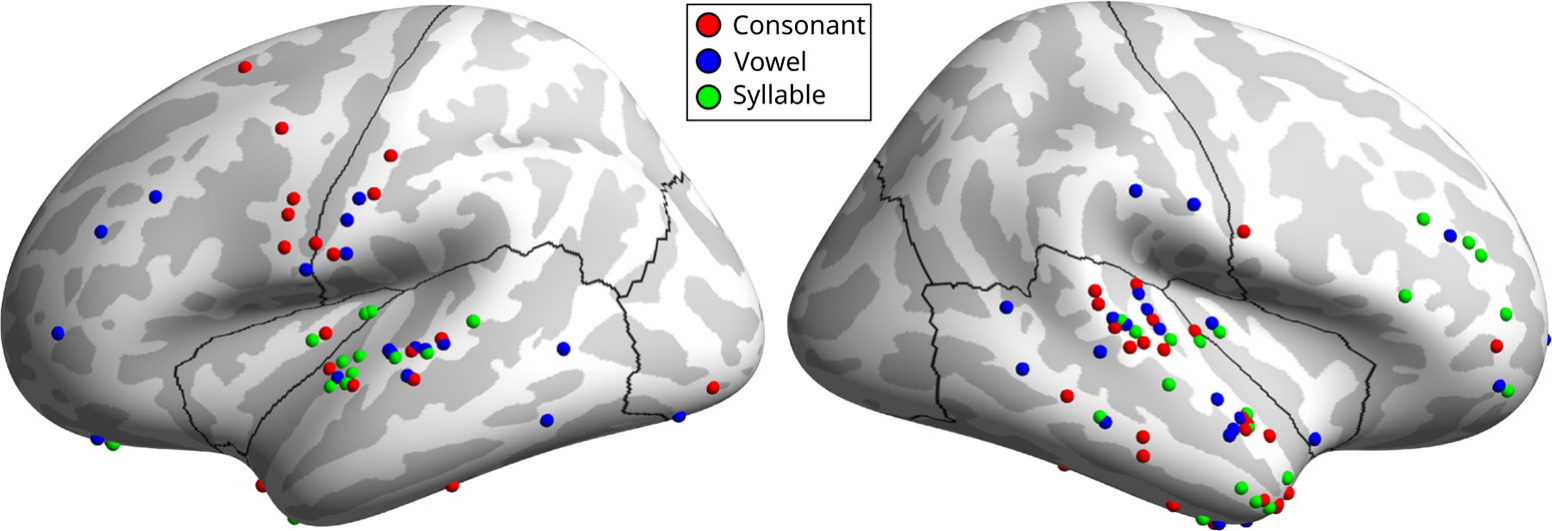
Electrodes with preferential encoding of consonant (red), vowel (blue), or syllable (green) compared with the other two phonemic units. For each electrode, classification was performed using only that electrode’s response for each of the three phonemic units. A bootstrap hypothesis testing procedure was then performed on resampled sets of trials to determine whether one of the three phonemic units had a significantly higher classification accuracy.

### Speech feature encoding and cluster order

To investigate possible functional roles of the electrode clusters, we examined how top-encoding electrodes of each speech feature were distributed across clusters (Fig. 6*B*). For consonant encoding, top electrodes were nonrandomly distributed (FDR-corrected *p* = 0.037, χ^2^), with increased probability of electrodes in the earliest and latest clusters (C1 and C6) and reduced representation in C4. Top-encoding electrodes for vowel did not show a bias for falling into any cluster (FDR-corrected *p* = 0.34, χ^2^). For syllable encoding, top electrodes were nonrandomly distributed (FDR-corrected *p* = 0.039, χ^2^). The syllable top-encoding electrode distribution showed an inverse distribution to consonant, with overrepresentation of syllable-encoding electrodes in C4 and reduced representation in earlier and later clusters.

### Speech feature encoding and response width

The encoding of speech features were compared with the response width of clusters, quantified as the time between onset and offset (see Table 2). Logistic regression was used to predict the number of top-encoding electrodes in each cluster from response widths (Fig. 6*C*). Proportion of top consonant encoders showed a nonsignificant positive trend with response width (FDR-corrected *p* > 0.050). Proportion of top vowel-encoding electrodes showed a nonsignificant negative trend with response width (FDR-corrected *p* > 0.065). Response width significantly predicted proportion of top syllable-encoding electrodes, with shorter-duration clusters containing a higher proportion of syllable-encoding electrodes (FDR-corrected *p* < 0.001).

### Classification using fixed or cluster-specific windows

We tested whether speech decoding performance would be affected by using neural activity from a cluster-specific time window, rather than the uniform 2-s window centered on speech onset used in the preceding analyses. In this modified procedure, decoding was performed for each cluster using activity in the time period when that cluster was most active (between “start” and “end” times in Table 2). A paired *t* test was used to compare subject-level decoding accuracy between the two analysis windows. Using a cluster-specific analysis window did not significantly change decoding accuracy for consonant (*p* = 0.87, 36.6% fixed; 36.3% cluster-specific), vowel (*p* = 0.48, 44.3% fixed; 42.0% cluster-specific), or syllable (*p* = 0.91, 14.5% fixed; 14.3% cluster-specific).

### Cortical sites with preferential encoding of phonemic units

We evaluated individual electrodes for preferential encoding of one of the 3 phonemic units (consonant, vowel, syllable). This analysis differed from the prior examination of top-encoding electrodes in that it aimed to discover electrodes which show a significantly different strength of speech encoding of one of the three phonemic units relative to the other two units, rather than absolute strength of encoding without reference to the other two units (see Materials and Methods). A bootstrap hypothesis testing procedure was performed in which trials were resampled 10,000 times, with each electrode’s relative accuracy in decoding the three phonemic units compared in each trial set. These distributions were then used to determine whether each electrode showed significantly different accuracy in decoding one of the three units; 44.0% of responsive electrodes showed a phonemic unit preference (Fig. 7). Consonant-preferring electrodes in the left hemisphere were found primarily in sensorimotor cortex and superior temporal gyrus. Consonant-preferring electrodes were also found throughout the right temporal lobe. Vowel-preferring electrodes did not show a clear localization to particular regions and were distributed across peri-Rolandic areas, temporal cortex, and prefrontal areas in both hemispheres. Syllable-preferring electrodes in the left hemisphere showed more regional specificity, being found almost entirely in the posterior superior temporal gyrus and neighboring insula. In the right hemisphere, syllable-encoding electrodes were found in a group around the middle frontal gyrus, as well as being distributed throughout the temporal lobe.

## Discussion

In this study, we aimed to understand how the timescale of spoken phonological units affects the time-course and localization of their representation in cortical activity. Using electrocorticography recordings from patients while they spoke CVC syllables, we investigated the location and temporal profile of the encoding of three phonological units: short consonants (plosives and affricates), vowels, and whole syllables.

### Temporal response clustering

We first used a novel clustering and Kalman filter-based trend analysis procedure, which revealed six clusters (Fig. 3). The majority of electrodes fell into clusters active during speech production (C3 and C4). Smaller clusters were active during speech-preparatory (C1 and C2) or later, possible auditory processing epochs (C5 and C6). One prior study has performed unsupervised temporal clustering of ECoG responses during speech production (Leonard et al., 2019). Key differences from the current study are that Leonard and colleagues, used a 2-s delay period between stimulus and GO cue, and that they derived clusters both from passive listening and speaking (Leonard et al., 2019; see their Fig. 4). These authors also only analyzed left hemisphere responses, while our recordings were balanced between left and right hemispheres. Despite these differences, some overlapping clusters were found across studies. These include a response starting up to 1 s before speech onset and peaking ∼200 ms after speech onset, with left-hemisphere locations predominantly in somato-motor cortex (our C4, Leonard and colleagues’ Cluster 5). Our latest-responding cluster (C6), with onset shortly after speech onset and duration ∼1 s, also resembles Leonard and colleagues’ Cluster 4; these responses likely relate to auditory monitoring of speech.

### Hemispheric specialization for linguistic unit duration

In agreement with theories positing that the right hemisphere processes speech at a slower timescale than the left hemisphere (Sidtis and Volpe, 1988; Poeppel, 2003; Lazard et al., 2012), we found a positive relationship between duration of phonological unit and rightward lateralization. Syllable encoding was right-lateralized, vowel encoding was bilateral, and consonant encoding was left-lateralized (Fig. 5*A*). Our finding of bilateral vowel encoding replicates the results of a prior ECoG study which used a similar CVC repetition task (Cogan et al., 2014). These results suggest that output of longer linguistic units is preferentially controlled by the right hemisphere, while articulation of shorter-timescale events, such as plosives, is preferentially managed by the left hemisphere. Extensive lesion studies support this conclusion, as they have shown that disorders involving impaired articulation are caused by lesions to left perisylvian cortex (Hillis et al., 2004; Richardson et al., 2012; Ripamonti et al., 2018). Control of longer suprasegmental (prosodic) speech features are instead impaired by damage to right cerebral cortex (Shapiro and Danly, 1985; Ross and Monnot, 2008). Heritable developmental speech disorders have also revealed this functional-anatomic dissociation. Members of the KE family, in whom mutations of the FOXP2 gene cause verbal dyspraxia (Lai et al., 2001), exhibit abnormalities in speech-evoked neural responses and cortical gray matter thickness exclusively in the left hemisphere (Vargha-Khadem et al., 1998, 2005). This left-hemisphere impairment does not, however, disrupt production or perception of prosodic intonation in these individuals (Alcock et al., 2000).

These functions are not exclusively lateralized in our findings, as ∼30% of strongly syllable-encoding or consonant-encoding electrodes were found in the hemisphere opposite to the overall trend. The fact that many of the right hemisphere, syllable-encoding electrodes were found in the temporal lobe and remained active around speech offset suggests that the speech processing performed by these electrodes includes auditory monitoring. This possibility aligns with imaging evidence which associates auditory error monitoring with perisylvian areas in the right hemisphere (Toyomura et al., 2007; Tourville et al., 2008; Niziolek and Guenther, 2013). Additionally, our localization results align with prior findings that vowel identity can be more robustly decoded from temporal lobe than somatomotor cortex activity during speech production (Markiewicz and Bohland, 2016; Conant et al., 2018) and auditory self-monitoring (Milsap et al., 2019).

### Timing of phoneme and syllable encoding

Most of the top syllable-encoding electrodes fell into C4 or C5, clusters with neural responses closely coinciding with utterance timing (Fig. 6*B*). Strongly consonant-tuned electrodes showed an opposite temporal pattern to syllable-encoding electrodes, preferentially falling into clusters active primarily before (C1 and C2) or after (C6) speech production. Top vowel-encoding electrodes showed a temporal pattern between consonant and syllable, being distributed evenly across clusters active throughout the trial time-course. This result may appear to run counter to models of speech production in which whole intended speech sequences are represented in working memory during speech preparation, followed by reading-out of individual phonological elements in structures controlling motor execution (Bohland et al., 2010; Hurlstone et al., 2014). However, it should be noted that many of the syllable-encoding C4 and C5 electrodes were in the right hemisphere. As previously mentioned, activity of these sites may represent monitoring for deviations from the expected sensory reafferent feedback during speech, rather than representations of the phonological sequence in working memory which directly control motor output.

Regarding left-hemisphere responses, a possible explanation for the low proportion of syllable-encoding electrodes during speech preparation is the relative simplicity of the task. Single-syllable repetition may not elicit robust representations of phonological sequences in working memory, as are required by tasks involving, e.g., turn-taking (Bögels et al., 2015; Castellucci et al., 2022), multisyllable recitation (Herman et al., 2013; Gehrig et al., 2019), or spontaneous description (Troiani et al., 2008; AbdulSabur et al., 2014). An additional consideration is that somatomotor cortex areas which are active during, rather than before, speech production may perform effectively at decoding whole syllables by representing lower-order speech variables. These populations might encode articulatory gestures (Grabski et al., 2012; Conant et al., 2018) or somatosensory patterns (Miyamoto et al., 2006; Bartoli et al., 2016) which are unique to whole syllables because of coarticulation, and thus more effectively differentiate whole-syllable than single-phoneme identity. Decoding of whole words exclusively from these Rolandic regions has been demonstrated with a neuroprosthetic implant (Moses et al., 2021).

This explanation is also supported by our finding of syllable-encoding electrodes in left primary motor and somatosensory cortex (Fig. 5*B*).

### Shorter neural responses encode longer phonological units

Comparison of response width to encoding showed that syllable encoding was associated with shorter-duration clusters (Fig. 6*C*). We found a trend for the opposite relationship with consonants, which were encoded more strongly by clusters with greater widths. These findings run contrary to an expectation that longer-duration neural responses would more strongly encode longer phonological units. However, it should be noted the shorter duration clusters (including C4 and C5) tended to have responses closely coinciding with speech production, during which syllable identity was strongly encoded. Thus, one interpretation is that clusters driving motor execution and online error monitoring have activation times tightly constrained to the period of speech production, compared with clusters subserving other functions. In this single-syllable repetition task, the former clusters more strongly encoded syllable identity, as discussed above.

### Preferential encoding of phonemic units across cortical regions

We performed speech feature classification using single-electrode responses to investigate cortical sites which show significantly stronger encoding for consonant, vowel, or syllable. Left ventral motor and somatosensory cortex contained many preferentially consonant-encoding electrodes, a smaller number of vowel-encoding encoding electrodes, and no syllable-encoding electrodes (Fig. 7). This result aligns with prior ECoG studies reporting localization of consonant encoding in activity in the left ventral precentral and postcentral gyri, with vowel-encoding activity localized to an overlapping but broader set of cortical areas (Pei et al., 2011; Lotte et al., 2015). Intrasurgical electrical stimulation of this region has also been closely associated with motor speech errors, unlike the linguistic and semantic errors associated with microstimulation of other perisylvian regions (Tate et al., 2014; Leonard et al., 2019).

Preferentially syllable-encoding electrodes in the left hemisphere were found almost entirely in the posterior superior temporal gyrus, and syllable-encoding electrodes accounted for half of all preferential electrodes in this region. Posterior STG (Superior Temporal Gyrus) is considered to be a higher auditory area (Friederici, 2012) with selective responses to speech sounds (Rimol et al., 2005; Chan et al., 2014) and boundaries between suprasegmental linguistic units, including syllables (Luo and Poeppel, 2007; Ding et al., 2016; Oganian and Chang, 2019). Our results align with a prior intracranial EEG study which found reliable encoding of heard syllable identity in this area, independent of lower-order acoustic features (Bouton et al., 2018). While many studies of posterior STG have elucidated its responses during passive listening or auditory detection tasks, it has also been shown to play an important role in speech planning (Hickok et al., 2000; Strijkers et al., 2017).

Right middle frontal gyrus also showed a disproportionately high representation of preferentially syllable-encoding electrodes. This encoding may be related to a previously described role of this region in speech error monitoring, suggested by feedback perturbation responses (Kort et al., 2013) and transcranial magnetic stimulation-induced increases in naming errors (Sollmann et al., 2014). Right MFG was not found to detectably encode syllable identity in a similar speech production study using fMRI (Markiewicz and Bohland, 2016), suggesting that detecting syllable identity encoding in this area requires the high temporal and spatial resolution provided by intracranial recordings.

## Synthesis

Reviewing Editor: Anne Keitel, University of Dundee Decisions are customarily a result of the Reviewing Editor and the peer reviewers coming together and discussing their recommendations until a consensus is reached. When revisions are invited, a fact-based synthesis statement explaining their decision and outlining what is needed to prepare a revision will be listed below. The following reviewer(s) agreed to reveal their identity: Blaise Yvert.

The reviewers and editor agreed that the manuscript presents original analyses on hemispheric lateralisation during pseudoword reading with potentially interesting results. However, the reviewers pointed out important issues and questions that should be addressed to improve the manuscript.  

Most importantly, we would like to highlight that some complimentary analyses are necessary (e.g. regarding electrode choices, analysis of lateralisation in participant with electrodes in both hemispheres), and that some methodological choices need to be better justified (e.g. to only use a high-gamma power frequency band).  

In addition, please replace the ethically outdated term “subjects” with “participants” throughout the manuscript. Also, please substitute bar graphs, which do not allow assessment of underlying distributions, with more suitable graphs, such as boxplots or violinplots, ideally including individual data points. 

Please find the detailed, unabridged reviewer comments below, to allow you to respond to them in a point-by-point manner.   

*** Reviewer comments *** 

Reviewer # 1 - Advances the Field 

This manuscript is the first to present a hemispheric lateralization index of neural activity underlying pseudoword reading. 

Reviewer # 1 - Visual Abstract 

The visual abstract are informative and readable. To my opinion, they do not support the conclusion proposed by the authors. 

Reviewer # 1 - All Comments 

This study uses Electroencephalography (ECoG) recordings to investigate the temporal and spatial patterns of neural activity involved in speech production. Five adults read aloud CVC pseudowords while their neural activity was recorded. A novel method was used to classify the neural activity recorded on each electrode, making it possible to specify the spatial and temporal course of the activity. Furthermore, the activity of each cluster was classified to identify the neural activity specific to consonants, vowels and syllables articulated. The authors then calculated an index of hemispheric lateralization for each cluster, based on the results of the classification.  

My opinion on the work done is positive. The manuscript is well written. The structure of the manuscript is clear and the ideas presented in the manuscript are clearly stated. However, I would like the authors to work on a few points:  

Regarding the use of the word “spoken,” it would be important for the authors to clarify that the participants were reading aloud written pseudowords rather than producing spontaneous speech. This could affect the interpretation of the results and it is important to be clear about the task that was performed.  

The issue of artefacts in the neural signal due to muscular activation of the articulators is a valid concern. It would be important for the authors to either address this issue or specify that the ECoG data was free of such artefacts. If there were artefacts present, it would be important for the authors to describe how they were resolved in order to accurately interpret the results.  

The choice to use only the high-gamma power frequency band could potentially limit the conclusions that can be drawn from the study. It would be important for the authors to justify this choice and explain how it does not affect the interpretation of the results.  

The conclusion stated in the abstract and discussion does not seem to fully reflect the results of the study. The link between the succession of processing steps and the lateralization of processing is not clear and it would be important for the authors to address this. The results show (1) a succession of processing steps, (2) a lateralized processing according to the features investigated (i.e., consonant, vowel, syllable). But the link between the succession of processing steps and their lateralation is too indirect to conclude that there is “a successive encoding of larger phonological units leading into speech production”.  

It would also be interesting for the authors to discuss the results in the context of previous research on lesions and dysfunctions (using the studies presented in the introduction), as this could provide additional insight into the implications of the study. 

Reviewer # 2 - Advances the Field

The manuscript provides an original finding about lateralization of consonants versus vowels encoding during speech production 

Reviewer # 2 - Software Comments

The code is said to be provided by the authors but the link is missing due to confidentiality of the review process. Yet the code is not necessary to review the manuscript 

Reviewer # 2 - All Comments

The manuscript presents an original analysis of cortical activity underlying production of short CVC syllables. The authors report that consonants are preferentially encoded by the left hemisphere before and after speech production, while the whole syllables are preferentially encoded by the right hemisphere during speech production, supporting the hypothesis that the left hemisphere is preferentially engaged in the production of sharp speech elements, while the right hemisphere preferentially encodes speech features at longer time scales. The paper is very clear and well written. The analysis is clever and original. I only have the following comments to strengthen the manuscript and confirm the conclusions drawn by the authors.

Main comments:

1) The authors base their evaluation of the timing of speech feature encoding by considering the clusters to which the top electrodes belong. Yet, the LDA decoding uses all time points of the HG signal for each electrode. It is thus possible that some discriminative time points of a top electrode do not match the peak of the cluster to which this electrode corresponds. To confirm the different timing of encoding of the different speech features, it would thus be important to also have a complementary analysis of the latency of the discriminative features of each top electrode.  

2) Alternatively, the decoding procedure could only use the temporal neural features of each electrode around the peak latency of the cluster that the electrode belongs to.  

3) It is not clear whether the decoding results that are further used to infer the top electrodes are significantly above chance level. This chance level should thus be evaluated and only the decoding results with accuracy significantly above chance be further considered to infer the top electrodes.  

4) I am wondering whether the spatial coverage of top electrodes in Figure 4 differs across the 3 speech features. Could this be evaluated (maybe with a MANOVA)?  

5) One subject has electrodes on both hemispheres. What would give the analysis on this single subject? In particular, can the lateralization results of Figure 5 be observed?  

Further minor comments:  

1) I am not sure what objective approach was used to eventually consider 6 clusters. Please better explain how this was set and if the results would be replicated with another number of clusters  

2) Both ECoG and SEEG electrodes were used for the analysis. Could Figure 1 show each type with a different symbol to know which electrodes are on the cortical surface and which are deep? Also, if possible, on subsequent result figures.  

3) At the top of page 8 it is mentioned that the trend analysis selects 334 electrodes out of 1036. How many are on each hemisphere?  

4) Figure 3 lacks a legend for panel B  

5) Legend of Figure 6A: Supplementary Figure S1 not S2  

6) Error bars in Figure 6B are not clearly visible 

## Author Response

Reviewer comment:

“The fact that the right-lateralization of syllables becomes not statistically significant with top-60% electrodes is a bit worrying as it is presented as a main finding. I would thus recommend to maybe modulate accordingly the corresponding sentence in the abstract and also clearly mention in the results (lines 569-571) that this lateralization becomes a trend with top-60% electrodes. At the moment it is written that the results are similar, which is not exactly the same.”

Author Response:

The abstract has been edited to clarify that the ‘strongly encoding’ electrodes with hemispheric preferences were the top 30%. We believe that these results which focus on a minority of strongly encoding electrodes (top 30%) are more consequential than those which consider a larger proportion (top 60%) because of the nature of the test that was used to evaluate hemispheric preference. This test uses a binomial distribution to determine whether the proportion of right-hemisphere electrodes within a subset of all electrodes is significantly different from the proportion of right-hemisphere electrodes in the total electrode population. (This whole-population proportion - 55.3% right-hemisphere - is treated as ‘chance’ level.) Thus when 100% of electrodes are included in the subset, significant lateralization is impossible to find because the proportion of right-lateralized electrodes in the ‘subset’ is the same as chance level, by definition. Similarly, as the proportion of electrodes in the subset is increased (from 30% to 60%), the likelihood of null findings becomes larger, due to the increased overlap between the subset and total population.

We argue that the top-60% analysis is less relevant than the top-30% analysis because the top 60% analysis adds to the subset a number of electrodes which may only weakly encode the speech feature of interest, including some that have below-median encoding performance. Using the larger 60% subset was necessary when analyzing a single subject, due to the small number of total electrodes in one subject, but is not appropriate in the all subject analysis. However, we appreciate the critique that it was not accurate to state that the 60% and 30% results were ‘similar’ when only one was statistically significantly lateralized for syllable encoding. To avoid confusion, we have eliminated the description of the 60% analysis from the Results section titled ‘Lateralization of speech feature encoding’ when describing group level results.

(The Abstract has also been shortened to meet the 250 word limit.)
