# Supplementary figures and images for: Lateralization and Time-Course of Cortical Phonological Representations during Syllable Production
Source: eNeuro. 2023 Oct 5;10(10):ENEURO.0474-22.2023. doi: 10.1523/ENEURO.0474-22.2023 (PMC10561542; doi:10.1523/ENEURO.0474-22.2023)

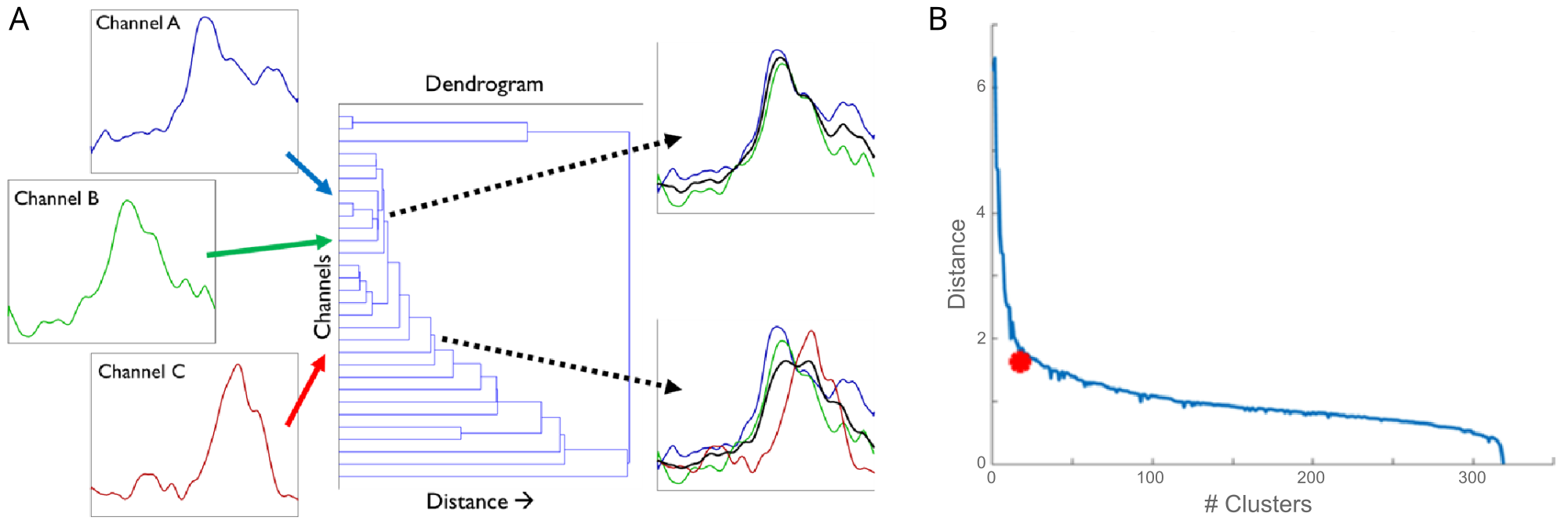

Supplement: Extended Data Figure 2-1 — Clustering dendrogram schematic and effect of cluster number on mean distance. A, Dendrogram illustration of cluster tree showing at what distance each channel gets merged into a cluster. Moving left to right, each channel is initialized as its own cluster and is iteratively merged until a single cluster remains. Temporal profiles of three example channels are plotted for illustrative purposes to depict when these channels would be clustered together. B, Relationship between number of clusters and the distance needed to result in a tree with that number of clusters. The red marker denotes the location that is selected for providing the number of clusters using the percentage of variance explained, which sets the distance threshold for selection. This point visually aligns with the “knee in the curve” heuristic. Download Figure 2-1, TIF file. [file enu-eN-NWR-0474-22-s02.tif]

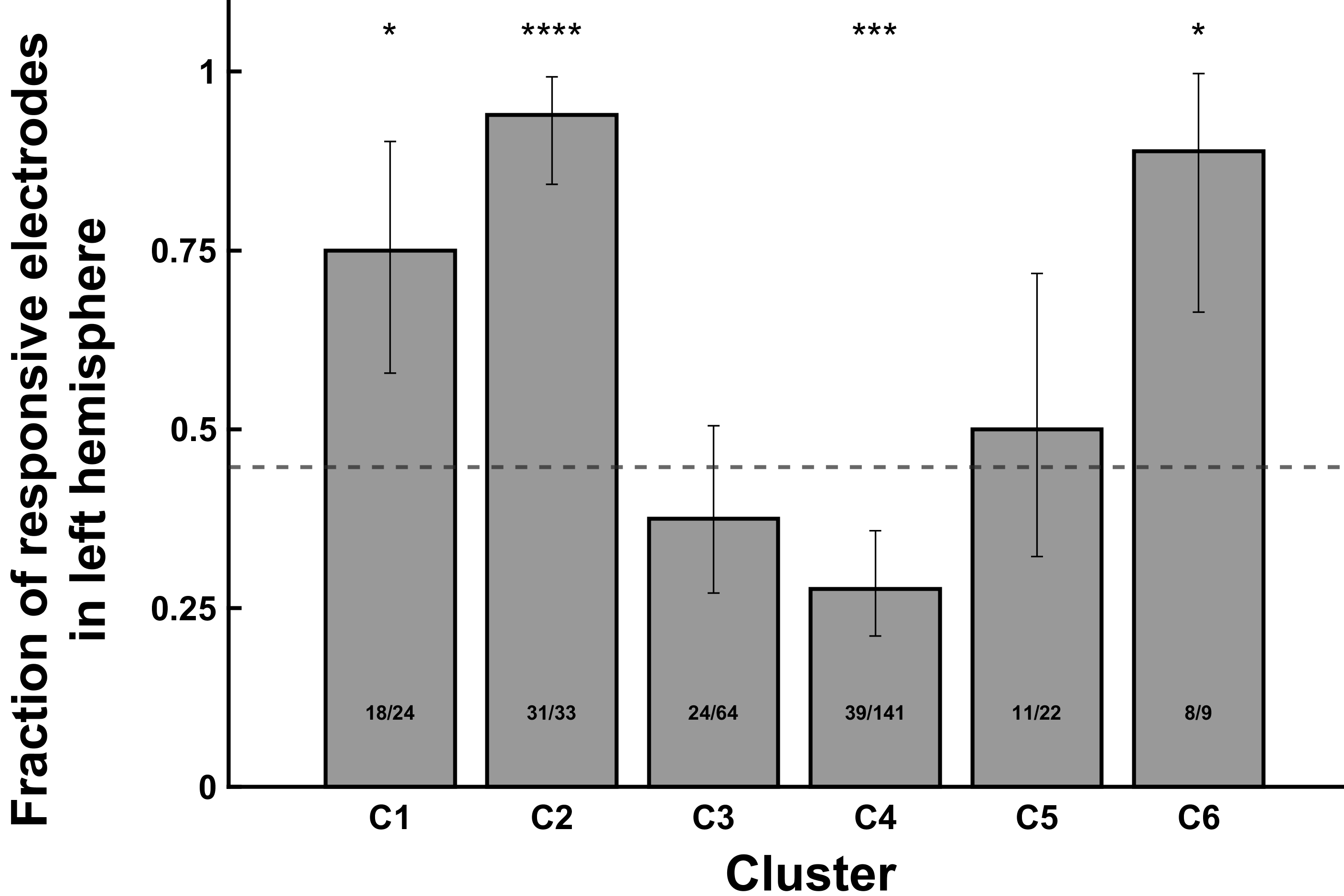

Supplement: Extended Data Figure 6-1 — Hemispheric lateralization of clusters, organized in order of high-gamma power response onset time. Bars show proportions of electrodes in each cluster in the right hemisphere. All responsive electrodes are included in this plot. (For similar analysis of including only top-encoding electrodes, see Fig. 6A.) Dotted horizontal line shows the chance proportion of right-hemisphere electrodes, determined as the proportion of all electrodes in the right hemisphere. Stars indicate deviations of individual clusters from chance proportion (FDR-corrected two-tailed binomial test). p-value stars indicate: *p = 0.05, **p = 0.01, ***p = 10−3, ****p = 10−4. Download Figure 6-1, TIF file. [file enu-eN-NWR-0474-22-s03.tif]
